# Supplementary material for: fosA11, a novel chromosomal-encoded fosfomycin resistance gene identified in Providencia rettgerihttps://blast.ncbi.nlm.nih.gov/Blast.cgi#alnHdr_343201689
Source: Microbiol Spectr. 2023 Dec 27;12(2):e02542-23. doi: 10.1128/spectrum.02542-23 (PMC10846113; doi:10.1128/spectrum.02542-23)
Supplement: Table S1 to S3 — Supplemental tables. [file spectrum.02542-23-s0001.docx]

**Table S1. Primers used in this study.**

| **Primers** | | | **Sequences (5′ to 3′)** | | **Product size** | |
| --- | --- | --- | --- | --- | --- | --- |
| *aac(2')-like*-*XbaI*-F  *aac(2')-like*-*BamHI*-R  *Spd-like-XbaI*-F  *Spd-like-BamHI*-R  *fosA11*-*XbaI*-F  *fosA11*-*BamHI*-R  *aph(3')-like*-*XbaI*-F  *aph(3')-like*-*BamHI*-R  *bla*_RSA_*-like*-*XbaI*-F  *bla*_RSA_*-like*-*BamHI*-R  *bla*_KPC_*-like*-*XbaI*-F  *bla*_KPC_*-like*-*BamHI*-R  *bla*_SRT_*-like*-*XbaI*-F  *bla*_SRT_*-like*-*BamHI*-R  *CblA-like*-*XbaI*-F  *CblA-like*-*BamHI*-R  *bla*_OXA335_*-like*-*XbaI*-F  *bla*_OXA335_*-like*-*BamHI*-R  *bla*_OXA59_-*like*-*XbaI*-F  *bla*_OXA59_-*like*-*BamHI*-R | | | GCTCTAGAATGACTATCCAATATTTCAGTCGCC  CGGGATCCTTACCACTGATCACCACTGCGAAAATC  GCTCTAGAATGAATTTTCTCAATCAATTTTTC  CGGGATCCTTAATGGCGATTTCTCACCAATTG  GCTCTAGAATGAGTACTGGGACGAAAAAGTTGTTCAC  CGGGATCCTTTAATAAAATTTCATACCTTCATAAGGGGC  GCTCTAGAATGGAAAACCCTTGGATTTTATCTC  CGGGATCCTTATTTCCCTGTAAACTGAGGTTTTC  GCTCTAGAGTGAAGAAAATGATTAGCGGGGC  CGGGATCCTCAGTAAGACATCGCTAATGCATTG  GCTCTAGAATGAATTGGTATCCTTGGTTAAATG  CGGGATCCTTAAAATGAAGGTGAAGCCAATAAG  GCTCTAGAATGAGAAATGTTTTTCGTCAGGGG  CGGGATCCTTATTCATTAATCTTTTCAATTAATTG  GCTCTAGAATGGACTTGTTCGCCTATGGTGC  CGGGATCCTCATTCTGCGACCTGTTTTTTGG  GCTCTAGAATGCAGGAATTTCCTTTAGTTTTTTTT  CGGGATCCCTAGAGGTGATTAAATGTAATTAAC  GCTCTAGAATGAGCACAATGGATAGATTGGAT  CGGGATCCTTAATTACTGGGGACCAGTAATAG | | 537 bp  678 bp  616 bp  774 bp  660 bp  993 bp  1143 bp  945 bp  828 bp  771 bp | |
| 16S rRNA-F  16S rRNA-R  *fosA11*-*XbaI*-F | | AGAGTTTGATCCTGGCTCAG  TACGGCTACCTTGTTACGACTT  GCTCTAGAATGAGTACTGGGACGAAAAAGTTGTTCAC | | | 1465bp  831bp | |
| *fosA11*-*BamHI*-R | CGGGATCCTTTAATAAAATTTCATACCTTCATAAGGGGC | | |  | |  |
| *fosA11* -F | TCTCCTGTGGGGATTTATGG | | | 235bp | |  |
| *fosA11* -R | CCACCCACATGCAGTTCTAA | | |  | |  |
| *fosA11*-*XhoI*-ORF- EK-F  *fosA11*-*HindIII*-ORF-R | CCCTCGAGGATGATGATGATAAGATGTTAGTTGGAATTAACCATTTAAC  CCAAGCTTTTTAATAAAATTTCATACCTTCATAAGGGGC | | | 445bp | |  |
| *fosA11-*mut7 -F | AGTTGGAATTAACGCGTTAACCATTGCAG | | | 362bp | |  |
| *fosA11* mut7-R | CACTGCAATGGTTAACGCGTTAATTCCAAC | | |  | |  |
| *fosA11-*mut67 -F | AACAGATTACACTGCGTATGCTTTTACAG | | | 471bp | |  |
| *fosA11* mut67-R | TAACTGTAAAAGCATACGCAGTGTAATCTG | | |  | |  |
| *fosA11-*mut113-F | ATGGGCACAAATTAGCGCTGCATGTGGGTG | | | 812bp | |  |
| *fosA11* mut113-R | AAGCCACCCACATGCAGCGCTAATTTGTGCCC | | |  | |  |

**Table S3. Antimicrobial susceptibility test results for *Providencia rettgeri* W986 and recombinant strains harboring candidate resistance genes (mg/L).**

|  | Fosfomycin*^a^* | Gentamicin | Streptomycin | Amikacin | Tobramycin | Ampicillin | Cefuroxime | Cefixime |
| --- | --- | --- | --- | --- | --- | --- | --- | --- |
| *E. coli* ATCC25922 | 1 | 0.5 | 4 | 1 | 0.5 | 4 | 2 | 0.06 |
| *Providencia rettgeri* W986 | 1024 | 0.5 | 512 | 0.5 | 1 | 1024 | 1024 | 8 |
| pUCP20*/*DH5α | 1 | 0.25 | 1 | 1 | 0.5 | >1024 | >1024 | 0.5 |
| pUCP24*/*DH5α | 1 | 64 | 1 | 1 | 0.5 | 4 | 4 | 0.25 |
| *aac(2')-Ia/*pUCP20*/*DH5α | 1 | 0.25 | 1 | 1 | 0.5 | >1024 | >1024 | 0.5 |
| *aph(3')-IX/*pUCP20*/*DH5α | 1 | 0.25 | 1 | 1 | 0.5 | >1024 | >1024 | 0.5 |
| *srt/*pUCP20*/DH5α* | 1 | 32 | 1 | 0.5 | 0.5 | 4 | 4 | 0.5 |
| *fosA11/*pUCP20*/*DH5α | 256 | 0.25 | 0.5 | 1 | 0.5 | >1024 | >1024 | 0.5 |
| *rsa-2/*pUCP24*/*DH5α | 1 | 32 | 1 | 1 | 0.5 | 4 | 4 | 0.5 |
| *kpc-15/*pUCP24*/*DH5α | 1 | 32 | 1 | 0.5 | 0.5 | 4 | 4 | 0.5 |
| *spd/*pUCP24*/*DH5α | 1 | 64 | 1 | 1 | 0.5 | 8 | 4 | 0.5 |
| *oxa/*pUCP24*/*DH5α | 1 | 32 | 1 | 1 | 0.5 | 4 | 4 | 0.5 |
| *cblA-1/*pUCP24*/*DH5α | 1 | 32 | 1 | 1 | 0.5 | 4 | 4 | 0.5 |

*^a^* Fosfomycin with 25 μg/mL glucose 6-phosphate (G6P) according to CLSI standard.

**Table S2.** Antibiotic resistance phenotypes for *Providencia rettgeri* W986 (μg/mL).

| **antibiotics** | **W986** | **antibiotics** | **W986** | **antibiotics** | **W986** |
| --- | --- | --- | --- | --- | --- |
| Chloramphenicol | 32 | Tetracycline | 32 | Kanamycin | 64 |
| Florfenicol | 16 | Tigecycline | 0.5 | azithromycin | 1 |
| Ciprofloxacin | 0.5 | Cefepime | 128 | Spectinomycin | 128 |
| Fosfomycin | 1024 | Ceftazidime | 1 | Streptomycin | 64 |
| Levofloxacin | 32 | Cefotaxime | 128 | Neomycin | 2 |
| Polymycin E | >64 | Aztreonam | 256 | Sisomicin | 32 |
| Polymycin B | >64 | Ampicillin | >1024 | Ribostamycin | 16 |
| Gentamicin | 0.25 | Imipenem | 0.25 | Tobramycin | 64 |
| rifampicin | 4 | meropenem | 0.25 | tetracycline | 0.5 |
| Amikacin | 4 | Trimethoprim | 1 | Micronomicin | 64 |

**Sequence data**

The nucleotide sequence and amino acid sequence derived for the *fosA11* gene (GenBank accession no. MZ277617.1) are provided below for evaluating the manuscript.

>Complete nucleotide sequence of the *fosA11 gene*

ATGAGTACTGGGACGAAAAAGTTGTTCACATTAACTGTGGATAACCCTCAGTTTCCTTTGTGAATAACTCAATTAACTTTATTAATAACATAGACATATGTGGATGAGCTAAAAGTATTCAATTATAATAGGCAAAAAAAAACCTTTTTATGGGGGTCATAAAAAGGTTAAGGGCATCTATTAACAGGTTGTCTTAATGTTGATGTGTTATAGATTGTAACGGTTACAATGTTATTTACTGGTAGTTTATGTAAATTCATTAAAAGAAATCGTTTAGCGTTCATATAGGATGAAAAATAGTATGTTAGTTGGAATTAACCATTTAACCATTGCAGTGACTGATGTTGAGAAAAGCATCTTCTTTTATCAATCATTACTTGGTATGAAGCTACATGCAAGTTGGAAAAATGGAGCGTATATCTCCTGTGGGGATTTATGGTTATGTTTATCGTTAGATAAAACACGCTTATCTTTTTCACATACCGAAACAGATTACACTCATTATGCTTTTACAGTTAGTGAGGCAGATTTTCCTATCTGTGTTGCCAAACTAAAACAGGCTAATGTCATTGTTTGGAAAGAAAATAAGAGTGAGGGGAAATCATTTTATTTTCTTGACCCTGATGGGCACAAATTAGAACTGCATGTGGGTGGCTTATTGCAACGTTTAAAAAGCTGCCAAGAAGCCCCTTATGAAGGTATGAAATTTTATTAAATAAGCCTGAGAATTTCTTTACAAATGATTTTTAATTTTAAAAATAGCTTTACGTAGAGTGATATATCCTATTTTTAATGAATTATTAAAGAAATAGGAA

>Complete amino acid sequence of FosA11

MLVGINHLTIAVTDVEKSIFFYQSLLGMKLHASWKNGAYISCGDLWLCLSLDKTRLSFSHTETDYTHYAFTVSEADFPICVAKLKQANVIVWKENKSEGKSFYFLDPDGHKLELHVGGLLQRLKSCQEAPYEGMKFY*
